# Housing Context and Social Networks Among Lower Income Older Adults

**DOI:** 10.1093/geroni/igaa057.2505

**Published:** 2020-12-16

**Authors:** Noah Webster

**Affiliations:** University of Michigan, Ann Arbor, Michigan, United States

## Abstract

Housing context among lower income older adults plays an important role in shaping access to resources and ultimately well-being. We know very little about how housing influences access to social resources. This study examines the association between housing context (multi-unit vs. free-standing homes) and network structure among a U.S. nationally representative sample of independent living, lower income (<$15,000 in past year) adults age 65+ from the National Health and Aging Trends Study (N=1,795). Regression analyses indicate the housing-networks link is moderated by martial/partner status and gender. Among those married/living with a partner, living in multi-unit buildings (compared to free-standing homes) is associated with larger networks (i.e., more people to talk with about important things). Among women, living in multi-unit buildings was associated with more friends and neighbors in one’s network. Findings highlight variation in access to social resources across housing contexts. Findings should inform policy aimed at reducing social isolation.

